# Dissecting T cell or antibody immunodominance in a complex host-pathogen system

**DOI:** 10.1186/1475-2875-11-S1-O23

**Published:** 2012-10-15

**Authors:** Carla Proietti, Lutz Krause, Joanne Roddick, Angela Trieu, Denise L Doolan

**Affiliations:** 1 Queensland Institute of Medical Research, Herston, QLD 4029, Australia

## Background

Vaccines are considered the most cost-effective public health measure for the prevention of infectious diseases, with many demonstrated successes. However the *Plasmodium* parasite, causative agent of malaria, has eluded decades of efforts aimed at developing an effective intervention. Contributing to this is the complexity of the parasite life cycle and our poor understanding of the mechanisms and antigenic targets of host immunity to the parasite.

## Materials

To enhance our understanding of the host-parasite relationship and facilitate rationale vaccine design, we have generated independent proteome-wide datasets of antibody responses and T cell responses to *P. falciparum.* Parasite antigens have been prioritized on the basis of the frequency and magnitude of immune responses in individuals either experimentally infected or naturally exposed to malaria. We are integrating these complex datasets to develop metrics of immunological, structural and genetic parameters associated with antigen immunodominance. A range of computational tools and comparative genomic analyses have been applied to extract information on putative structural and functional features associated with both immunodominance and antigenicity. These analyses have also taken into account information on gene and protein features, transcript and protein expression, protein localization, post-translational modifications, and sequence conservation between different *P. falciparum* strains and *Plasmodium* species.

## Results

Our data demonstrate that the most antigenic parasite-encoded molecules are not randomly distributed throughout the proteome since a large number of potential antigens are not recognized. Importantly, we establish that the antigens sets that are highly reactive for T cells are distinct from those that are highly reactive for antibodies. Additionally, while antigens recognized preferentially by antibodies are highly polymorphic, the most T cell reactive antigens are highly conserved amongst *P. falciparum* strains and different *Plasmodium* species. This observed lack of epitope polymorphism contrasts with dogma in the field that important antigens or epitopes are polymorphic as a consequence of parasite evasion of host immune responses.

Further analyses are underway to determine the localization and the expression kinetics of immune targets and to identify any structural and functional characteristics associated with the relative immunogenicity of *P. falciparum* antigens or epitopes.

## Conclusions

This study represents, to the best of our knowledge, the first attempt to quantitatively and qualitatively define the parameters of immunodominance in humans in response to a complex pathogen. These data will facilitate the rational design of vaccines against malaria and provide the foundation for similar studies of other pathogens that threaten public health.

